# The Effects of User Engagements for User and Company Generated Videos on Music Sales: Empirical Evidence From YouTube

**DOI:** 10.3389/fpsyg.2018.01880

**Published:** 2018-10-05

**Authors:** JiHye Park, JooSeok Park, JaeHong Park

**Affiliations:** School of Management, Kyung Hee University, Seoul, South Korea

**Keywords:** user engagement, user-generated contents, company-generated contents, youtube, video, music sales

## Abstract

With the growth of social network services, users have been able to freely create and share music in ways that were once thought unimaginable. Sharing a music video through such platforms can now be done simply by anyone with access to a computer or smart phone. Online music content can be divided into two major categories—user-generated content (UGC) and company-generated content (CGC). While previous studies on the content of online music videos have examined the impact of this content on, for example, a company's marketing effectiveness and brand image, they have given little attention to the different effects of user engagement of UGC and user engagement of CGC. This study attempts to address this lack by examining how these two kinds of user engagement differently influence user music choices. We will also examine the differing levels of impact on users across the initial, middle, and final periods after a song is released. In order to examine the different impacts of user engagement, we apply an estimation of generalized least squares (GLS) with a panel dataset of 1,035 songs found on Gaon, the official South Korean music ranking chart. We use the number of music video shares generated by users and firms as proxy variables of user engagement in UGC and CGC, respectively. We find that user engagement of UGC and CGC positively influence music sales. We also find that the effects are not static, but rather change in the initial, middle, and final periods after a song is released. In particular, this study finds that the effect of user engagement of UGC on sales is greater than the effect of user engagement of CGC in the initial period but that the effect of the latter becomes similar to that of former at the end of this period. This finding suggests that managers of digital music providers should encourage consumers to create their own content in the initial period, as potential consumers are more likely to buy songs with more UGC content and shares in the initial period.

## Introduction

YouTube, one of the most famous music platforms in the world, has redefined the way we share music. We can like, share, or even upload videos of our favorite artists, giving friends a look into our musical preferences while at the same time publicizing artists that would have otherwise remained unknown. While YouTube has played a crucial role in the international proliferation of K-pop over the last decade, it also provides the stage on which new K-pop stars rise or fall in the Korean domestic market, before their names and songs reach trending lists abroad. On August 27, 2014, a Korean girl group named EXID released their single “Up Down (Wi-Arae),” but it failed to attract public attention, and activity died down within a month. When it seemed that they had encountered a dead end, an anonymous fan uploaded a video of their performance on YouTube, and it reached more than 20 million views, shooting the group into the spotlight, ultimately leading them to top the national music broadcast rankings. The simple act of uploading a video by one fan, who had no relation to any music agency, was the first ripple in the process of EXID becoming one of the most popular recent music groups in South Korea.

In the digital music content industry, people freely create and share music through social network services (SNS; Peitz and Waelbroeck, 2006; Dewan and Ramaprasad, 2014). Sharing a music video on a social media platform can now be done with a few clicks of a mouse. Online music content that users can share can be divided into two types—user-generated content (UGC) and company-generated content (CGC)^1^. While previous studies on online music content have examined how the use of social media influences marketing effectiveness, a company's brand image, and its performance (e.g., Dewan and Ramaprasad, 2009, 2014; Dhar and Chang, 2009; Chi, 2011; Rui et al., 2011; Chen et al., 2014; Berlin et al., 2015), they have yet to address the question of how user engagement of UGC and user engagement of CGC differently influence user music product choices. To address this question, we first need to measure user engagement of UGC and CGC individually. We directly collect user behavior data from the Websites with an R program and use the number of shares of UGC and CGC as proxies of user engagement variables (Lee et al., 2013). Ultimately, this study attempts to use a novel methodology to measure user engagement, a psychological dimension, and show how the impact of such engagement changes over time.

In the fields of social psychology and marketing, the term “engagement” refers to both involvement and participation (Hwang and Thorn, 1999). Such engagement is often regarded as a form of business communication between consumers and a brand. As a result of social media, however, firms are increasingly losing while individuals are increasingly gaining the power to distribute information (Eisingerich and Bell, 2008). Therefore, leveraging user engagement of both UGC and CGC is critical for a firm's success in a technologically connected society. While previous studies have shown that user engagement is positively related to firm performance, we believe that this is the first study to examine how the impact of user engagement of UGC and CGC on user music product choices changes over time.

To investigate the different effects of user engagement of UGC and CGC in social media, we focus on music videos on YouTube. Music video content on YouTube is comprised of two major types of videos—UGC and CGC. We can easily see how users engage differently with the UGC and CGC by seeing how they share them in different periods. In particular, we believe that music videos are more effective and influential on social media platforms than other types of media used by marketers delivering the content, such as review texts, images, and voices. This study will therefore empirically investigate how user engagement of the two different types of music videos influence music sales. We will also examine when the effects of user engagement of UGC and CGC become more influential across the initial, middle, and final periods after a song is released. With a statistical and rigorous approach to a large size of user behavior data, this study suggests a novel measurement of user engagement and shows how these two kinds of user engagement differently influence user music choices over time.

The rest of the paper is organized as follows. The next chapter presents the literature review and research background. Subsequently, we explain our data source and our empirical model. We conclude by presenting some implications of our study and discussing some of the limitations of our research and some possible future research ideas.

## Literature review and background

### User engagement and firm performance

This study aims to supplement the literature on the relationship between user engagement and firm performance. According to Hwang and Thorn (1999), “the term ‘engagement' is used as a general term that refers to both involvement and participation.” Such engagement is often regarded as a form of business communication between consumers and brands in the marketing literature. As a result of the recent growth of user-generated content, however, businesses are increasingly losing their power to dictate their communication agendas (Eisingerich and Bell, 2008). Now, in our technologically connected society, user engagement has become a critical component in the success of a company or brand. Using meta-analysis, Hwang and Thorn (1999) found that, despite the differences among individual studies, an information system's success is positively correlated with user involvement and user participation. Various studies have investigated the relationship between human attitude (e.g., perceived value, motivation, satisfaction) and user engagement (Harter et al., 2004; Kim et al., 2013). These studies, however, were limited to user engagement of mass media and did not examine engagement of online social media (e.g., online music video contents).

### The effects of UGC and CGC on firm performance

UGC, known as user-created content (UCC), is a form of content created by users. UGC most often appears as supplements to online platforms, such as social media websites, and may include content such as blog posts, wikis, videos, or comments (Daniasa et al., 2010). UGC is used for a wide range of applications, including problem processing, news, entertainment, advertising, gossip, and research (Chin-Fook and Simmonds, 2011). Previous research on the use of social media has focused on the effectiveness of social media on improving a company's image and performance. These studies have focused on the relationship between the use of social media and a firm's performance. Such research observed that the use of social media influenced a company's performance and that, overall, a greater use of social media had a more positive impact on the company's performance. For instance, UGC in a marketing context has been known to enhance firm brand image in numerous ways (York, 2016), with research even showing that marketing activities incorporating UCG are as effective as other types of media (Battelle, 2016).

In studies on the role of UCG in music sales, Dhar and Chang (2009) found that an increase of blog posts on an album positively influenced album sales. Dewan and Ramaprasad (2009) also showed evidence of bi-directional causality for sales and buzz in the music industry. Blog buzz has a positive and significant relationship with album sales, a relationship that is stronger for music released by independent labels than by major labels. (Dewan and Ramaprasad, 2014) extended their previous study and examined the effects of both mass media (radio broadcasting) and social media (blog buzz) on music sales. Radio play is consistently and positively related to future sales at both the song and album levels. However, blog buzz is not related to album sales and is negatively related to song sales. The negative relationship between song buzz and sales is stronger for niche music relative to mainstream music as well as for less popular songs within albums. Frick et al. (2014) also found that volume has a significant and positive impact on physical sales for releases by independent labels, but not those by major labels, and that it is crucial for major label artists to actively participate in social media.

### User engagement of UGC

Recent studies have focused on the impact of the characteristics of content on user engagement of social media. Shin et al. (2016) use data from Tumblr to investigate the effect of company-generated posts on subsequent customer engagement (likes and re-bloggings). They focused on two main information sources: visual (images) and textual (text and tags) posts, and their results show that proper visual stimuli (e.g., beautiful images, adult-content, celebrities), complementary textual content, and consistent themes have positive effects on engagement. Visual and textual features were also shown to have heterogeneous effects at different perspectives (industry-level, hedonic/utilitarian products, followers/non-followers, short/long-term engagements). Lee et al. (2013) describe the effect of social media advertising content (company-generated massages) on customer engagement (likes, comments, shares, and clicks) using data from Facebook. Their results show that brand personality-related content, such as emotional and humorous content, is positively associated with higher engagement. They find that directly informative content is associated with lower engagement of social media but that certain types of informative content can increase the number of clicks. Liikkanen and Salovaara (2015) investigate how the characteristics of a video influence user engagement on YouTube. Their results show that there are notable differences in engagement between different types of music videos [traditional (CGC), user-appropriated (UGC), derivative (UGC)] and video genre (e.g., music, gaming).

### The impact of user engagement on sales

Unlike previous studies, our study will contribute to the related literature by investigating how user engagement of social media content, specifically music video content, influences music sales. We show that when users highly engage with both UGC and CGC videos, their engagement (e.g., sharing music videos) positively influences music sales. As UGC on social media platforms provides immediate feedback from users and is considered more trustworthy than CGC, we believe that the impact of engagement of UGC is greater than that of CGC (Gopal and Sanders, 2006).

We also observe that most social media advertisements are highly concentrated in the initial period after a song's release. As few people know about the song during this period, marketers may want to promote more heavily, resulting in their marketing efforts becoming more influential. Individual music consumers may also want to disseminate songs they like as quickly as possible them more popular. Such consumers often create UGC of the songs or artists they like and share this content on online social media platforms (e.g., YouTube) in the initial period. Little is known, however, about the dynamics of user engagement of UGC and CGC on music video choices over time. This research thus examines when the impact of user engagement of UGC and CGC becomes more influential across different periods after a song is released.

Based on the literature review and arguments above, we propose the following hypotheses:

*H1: User engagement of user-generated videos (UGC) has a greater impact on music than does user engagement of company-generated videos (CGC)*.
*H2-a: User engagement of user-generated videos (UGC) has a greater impact on music sales during the initial period after a song is released than during the middle and final periods*.
*H2-b: User engagement of company-generated videos (CGC) has a greater impact on music sales during the initial period after a song is released than during the middle and final periods*.

## Data

This study chose to focus on the digital music industry for the following reasons. First, it most actively uses social media (Dewan and Ramaprasad, 2014). Second, as the industry is a high-value industry, finding the factors that influence music sales is important for business management. Third, in this industry, new products are actively published and are sensitive to trends, and purchases of products occur frequently. Fourth, this industry has a reputable organization that releases sales records, which enables us to collect sales-related data.

**Figure 1:**
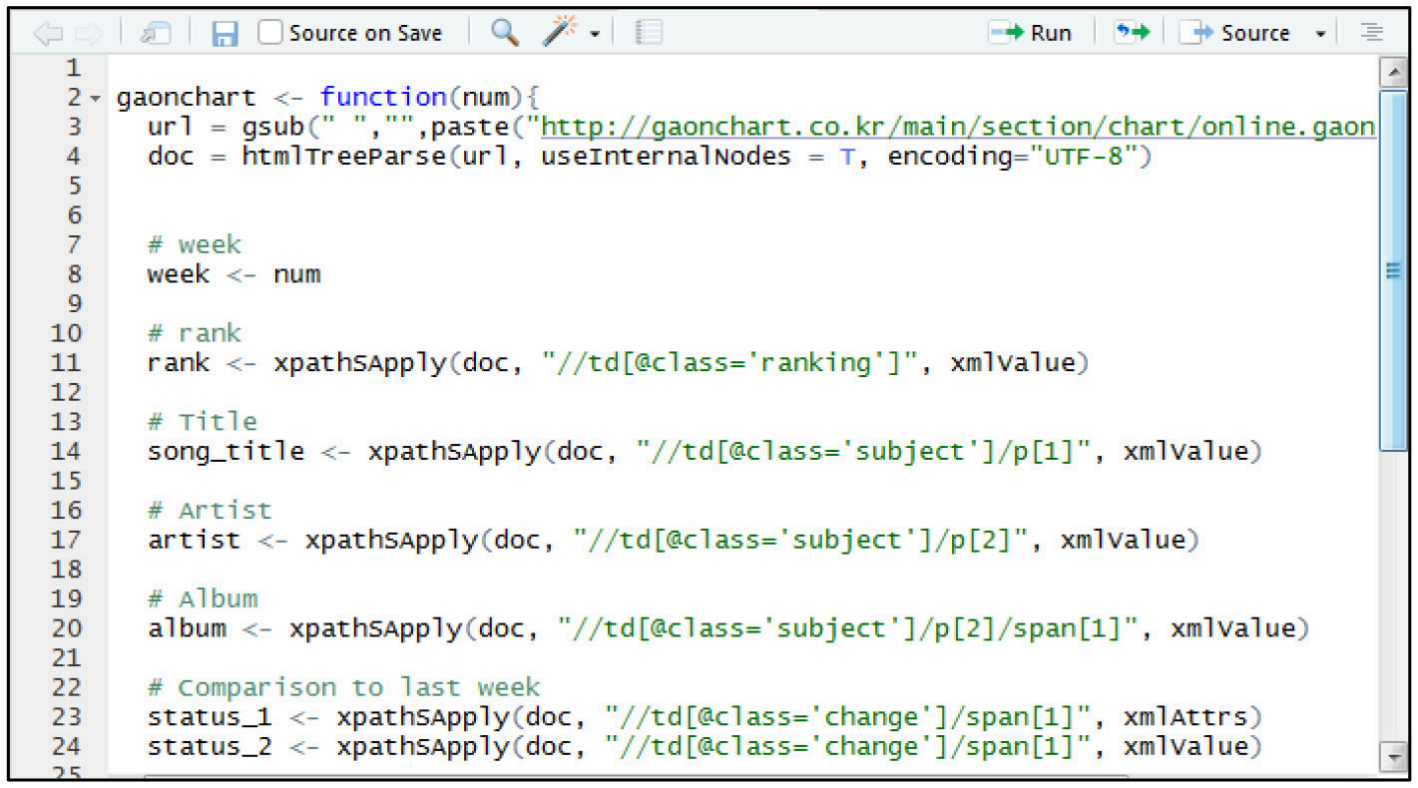
Example of our web crawling program.

We collected our data through the web-crawling method by using “R” on Gaon chart (www.gaonchart.co.kr), YouTube (www.youtube.com), and Melon (www.melon.com). “R” has an “Inspect Element” function provided by the webpage that allows for more effective data collection effective through many useful functions, so it is helpful for the data collection for this research. Figure 1 below is an example of the “R” program used to collect data from the websites. We first collected the music rank information from the Gaon Chart every week from December 27, 2015 to December 31, 2016. During this period, we had a total of 1,035 songs after removing song titles that were repeated. We then collected the music videos related to the 1,035 songs (e.g., UGC and UGC) from YouTube, which resulted in a total of 16,162 music videos.

Gaon Chart is the only official music chart in South Korea, and it collects data on the activities of 20 million monthly users. We collected music information (e.g., digital music sales rank, musician name, production firms, distributors) from Gaon on a weekly basis. YouTube is the largest video content sharing website in the world; it allows users to freely upload and watch video clips and enables them to share video content with others. Music producers, distributors, and entertainment companies have recently been managing YouTube accounts in order to advertise releases of new songs. Information regarding posting date, view count, share count, follower count, poster name, and the video titles were collected from YouTube. Other information, including the published date and genre of music, was gathered from Melon, one of the largest digital music distributors in Korea. Table 1 provides the list of our variables, and (Table 2) provides their descriptive statistics.

**Table 1:** Description of variables.

**Variables**	**Description**
*Sales*_*Rank*_*i, t*_	Song *i*'s sales rank at time *t*
*Num*_*of*_*UGC*_*Shares*_*i, t*−1_ [Proxy of user engagement of UGC]	The number of shares of videos generated by the user (of song *i*, at time *t*-1)
*Num*_*of*_*UG*_*CShares*_*i, t*−1_ [Proxy of user engagement of CGC]	The number of shares of videos generated by the company (of song *i*, at time *t*-1)
*Num*_*of*_*UGC*_*Shares*_*i, t*−1_×*Time*_*early*_*t*_	Interaction term between *Num*_*of*_*UGC*_*Shares*_*it*−1_ and *Time*_*early*_*t*_
*Num*_*of*_*UGC*_*Shares*_*i, t*−1_×*Time*_*middle*_*t*_	Interaction term between *Num*_*of*_*UGC*_*Shares*_*it*−1_ and *Time*_*middle*_*t*_
*Num*_*of*_*CGC*_*Shares*_*i, t*−1_×*Time*_*early*_*t*_	Interaction term between *Num*_*of*_*CGC*_*Shares*_*it*−1_ and *Time*_*early*_*t*_
*Num*_*of*_*CGC*_*Shares*_*i, t*−1_×*Time*_*middle*_*t*_	Interaction term between *Num*_*of*_*CGC*_*Shares*_*it*−1_ and *Time*_*middle*_*t*_
*Time*_*early*_*t*_	Dummy variable, if the number of days since song *i* was released is less than or equal to 14 days (2 weeks), then value = 1 (at time *t*)
*Time*_*middle*_*t*_	Dummy variable, if the number of days since song *i* was released is between 15 days and 35 days (5 weeks), then value = 1 (at time *t*)
*Num*_*of*_*clicks*_*i, t*−1_	The number of clicks of videos (of song *i*, at time *t*-1)
*Num*_*of*_*videos*_*i, t*−1_	The number of videos uploaded at time *t*-1 related to song *i*
*Num*_*of*_*subscribers*_*i, t*−1_	The number of subscribers into the YouTube accounts at time *t*-1 through the videos of song *i*
*Num*_*of*_*Days*_*After*_*Release*_*i, t*_	The number of days since song *i* was released at time *t*
*u*_*i*_	Fixed effect of each song *i* (unobserved time-invariant individual effect)
ε_*it*_	Error term

**Table 2:** Descriptive statistics of variables.

**Variables**	**Obs**	**Mean**	**Std.dev**	**Min**	**Max**
*Sales*_*Rank*_*i, t*_	4,752	49	28.71	1	100
*Num*_*of*_*UGC*_*Shares*_*i, t*−1_	4,752	229	1,660.26	0	69,840
*Num*_*of*_*UGC*_*Shares*_*i, t*−1_	4,752	2871	12,300.59	0	288,610
*Time*_*early*_*t*_	4,752	0	0.41	0	1
*Time*_*middle*_*t*_	4,752	0	0.40	0	1
*Num*_*of*_*clicks*_*i, t*−1_	4,752	561,683	1,652,171.00	1	45,300,000
*Num*_*of*_*videos*_*i, t*−1_	4,752	194	474.99	1	12,581
*Num*_*of*_*subscribers*_*i, t*−1_	4,752	234	1,135.98	−2	36,059
*Num*_*of*_*Days*_*After*_*Release*_*i, t*_	4,752	167	538.78	2	6,462

The dependent variable in this research is measured by the sales rank of song *i*, which represents music sales. Following previous research, this study also measures user engagement of the music video by the number of shares (Lee et al., 2013).

**Figure 2:**
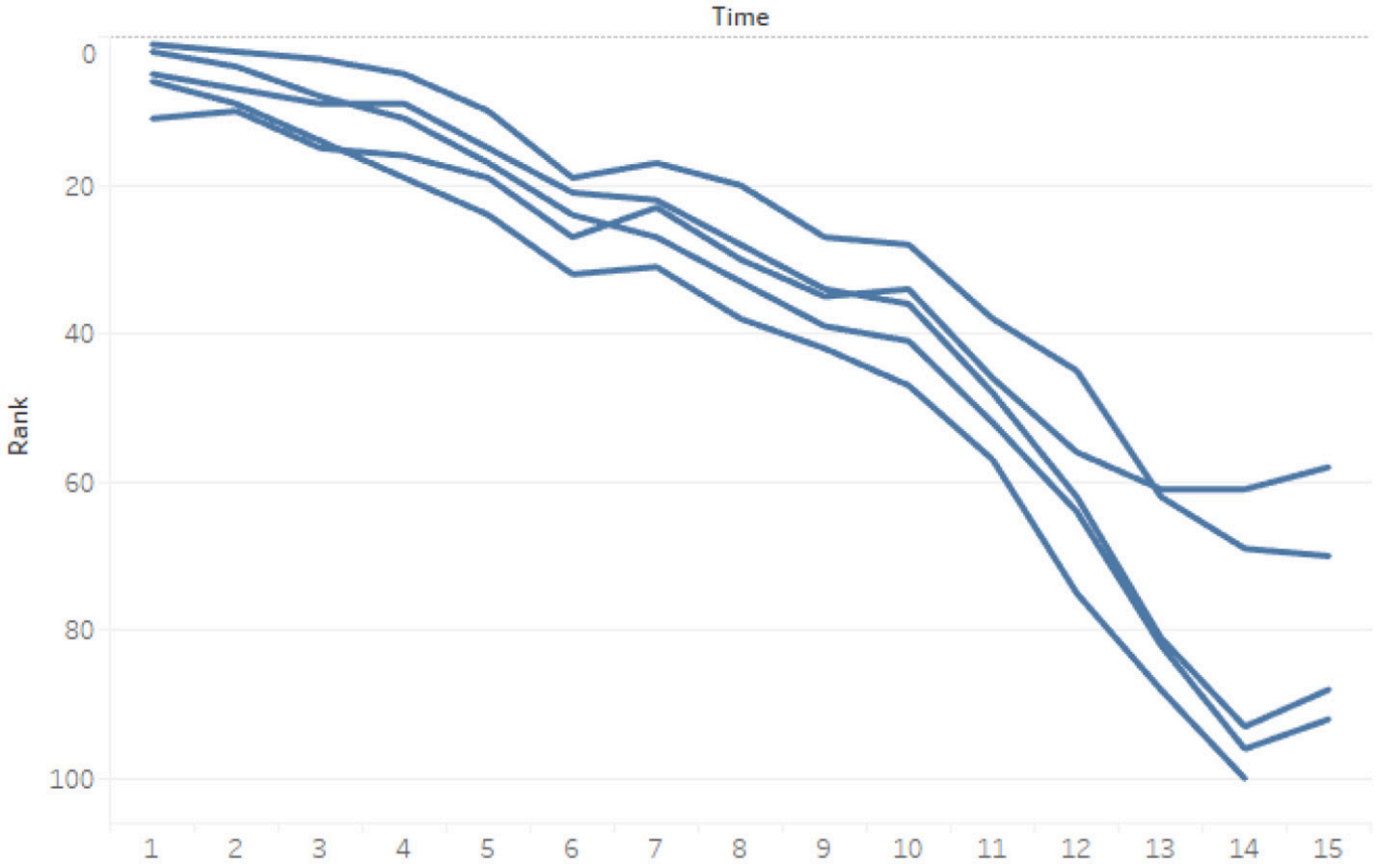
Example of sales ranks of songs.

We divide the lifetime of the music video into three periods—initial (between the 1st day and 14th day (2 weeks) after the video's release), middle [between the 15th day and 35th day (5 weeks)], and final (after the 36th day)—according to its diffusion characteristics. The 1,035 songs in this study appeared on the Gaon Chart for an average of ~5 weeks (35 days), and most of the songs' sales ranks (95%) were the highest in their first or second week on the chart. The songs' sales ranks decreased slowly starting from the second to the fifth week, after which they decreased rapidly. Figure 2 shows the diffusion characteristics of these songs.

Table 3 reports the correlations among key independent variables. As some correlations are comparatively high, we checked the VIF values of independent variables to avoid possible multicollinearity. VIF values of all independent variables were found to be less than 5.69, indicating that multicollinearity was not a serious problem for our analysis.

**Table 3:** Correlation of key variables (standardized).

**Variable**	**[1]**	**[2]**	**[3]**	**[4]**	**[5]**	**[6]**
[1] *Num*_*of*_*UGC*_*Shares*_*i, t*_	1.000	–	–	–	–	–
[2] *Num*_*of*_*CGC*_*Shares*_*i, t*_	0.389	1.000	–	–	–	–
[3] *Num*_*Of*_*clicks*_*i, t*_	0.663	0.894	1.000	–	–	–
[4] *Num*_*Of*_*videos*_*i, t*_	0.646	0.181	0.573	1.000	–	–
[5] *Num*_*Of*_*subscribers*_*i, t*_	0.447	0.761	0.896	0.307	1.000	–
[6] *Num*_*Of*_*Days*_*After*_*Release*_*i, t*_	−0.011	−0.05	−0.06	0.020	−0.04	1.000

## Model specifications

To see how user engagement of UGC and CGC influences music sales, we used the fixed-effect model. The fixed-effect model has the advantage of controlling for the characteristics of individual songs, which are consistent throughout. The data of this research is longitudinal data, which is cross-sectional and time-series at the same time. Longitudinal data generally has heteroscedasticity and auto-correlation issues. We conducted the likelihood-ratio test and the Wooldridge test to judge whether or not heteroscedasticity and first-order autocorrelations exist in our panel data. The results show that the data includes both heteroscedasticity (LR *chi*^2^ = 7,424.35, *p* = 0.000) and first-order autocorrelations (*F* = 361.143, *p* = 0.000). The GLS model is simple but robust under heteroscedasticity and autocorrelation circumstances, which is why we selected the panel GLS model to test our research model. We also added the song fixed effects into our GLS model to capture the idiosyncratic attributes of each song. We thus apply the panel generalized least squares (GLS) estimation to test our hypotheses. We also use a log-linear model to measure a percent change in our variables, and all of the continuous variables are log-transformed.

### Research model

(1) $$\begin{array}{l} \ln(Music\_Sales_{i,t})\\ \;=\beta_{1}\ln(Num\_of\_UGC\_Shares_{i,t-1}) \end{array}$$

(2) $$\;+\beta_{2}\ln(Num\_of\_CGC\_Shares_{i,t-1})$$

(3) $$\;+\beta_{3}\ln(Num\_of\_UGC\_Shares_{i,t-1})\times Time\_early_{t}$$

(4) $$\;+\beta_{4}\ln(Num\_of\_UGC\_Shares_{i,t-1})\times Time\_middle_{t}$$

(5) $$\;+\beta_{5}\ln(Num\_of\_CGC\_Shares_{i,t-1})\times Time\_early_{t}$$

(6) $$\;+\beta_{6}\ln(Num\_of\_CGC\_Shares_{i,t-1})\times Time\_middle_{t}$$

$$\;+\beta_{7}Time\_early_{t}+\beta_{8}Time\_middle_{t}$$

$$\;+\beta_{8}\ln(Num\_of\_clicks_{i,t-1})+\beta_{9}\ln(Num\_of\_videos_{i,t-1})$$

$$\;+\beta_{10}\ln(Num\_of\_subscribers_{i,t-1})$$

$$\;+\beta_{11}\ln(Num\_of\_Days\_After\_Release_{i,t})+u_{i}+\varepsilon_{it}$$

Let *i* = 1, …, N index the songs. The dependent variable, *Music*_*Sales*_*i, t*_, denotes the log value of song *i*'s sales rank at time *t*. In terms of ranking, a large number means a low rank while a small number means a high rank, which may cause confusion in interpretation. The rank is thus multiplied by minus one after log-transform to interpret the regression coefficients more intuitively as changing the sign of the variable. *Num*_*of*_*UGC*_*Shares*_*i, t*−1_ is one of the key variables that capture the impact of user engagement of user-generated videos (UGC) on music sales. *Num*_*of*_*CGC*_*Shares*_*i, t*−1_ is another key variable that captures the impact of user engagement of company-generated videos (CGC) on music sales. β_1_ and β_2_ captures how user engagement in UGC and CGC influences music sales.

We then added interaction terms to measure the interaction impact between user engagement and song diffusion after their release. *Num*_*of*_*UGC*_*Shares*_*i, t*−1_×*Time*_*early*_*t*_ and *Num*_*of*_*UGC*_*Shares*_*i, t*−1_×*Time*_*middle*_*t*_ represent the interaction effect between user engagement for UGC and a song's diffusion on music sales (the base is the final period). Also, *Num*_*of*_*CGCShares*_*i, t*−1_×*Time*_*early*_*t*_ and *Num*_*of*_*CGC*_*Shares*_*i, t*−1_×*Time*_*middle*_*t*_ represent the interaction impact between user engagement for CGC and a song's diffusion on music sales (the base is the final period). β_3_ and β_5_ show whether user engagement of UGC has a greater impact on music sales than user engagement of CGC in the initial period compared to the last period. Likewise, β_4_ and β_6_ examine whether user engagement of UGC has a greater impact on music sales than user engagement of CGC in the initial period compared to the last period.

We also include the following control variables. First, *Num*_*Of*_*clicks*_*i, t*−1_, *Num*_*Of*_*videos*_*i, t*−1_, and *Num*_*Of*_*subscribers*_*i, t*−1_ are included to capture the idiosyncratic attributes of songs. Second, the time-invariant individual song *i*'s fixed-effect term (*u*_*i*_) in our research model is included to control for each characteristic of song *i* (e.g., artist, distributor, production company). Third, *Num*_*Of*_*Days*_*After*_*Release*_*i, t*_ is included to control for the different diffusion processes for each song.

## Results

We conduct a panel GLS estimation to control for both heterogeneity and autocorrelation using the balanced weekly panel data. The results are shown in Table 4. First, we estimate a model including only control variables (Column 1). Second, we estimate another model including only the main effects (Column 2 and 3). Third, we estimate models that include individual interaction terms (Column 4, 5, 6, and 7). Column 7 is the estimates of our final model.

**Table 4:** Estimation results.

**Variables**	**(1) Only control variables (C.V)**	**(2) C.V with main effect 1**	**(3) C.V with Main effect 1,2**	**(4) C.V with main effect 1,2 and interaction effect 1**	**(5) C.V with Main effect 1,2 and interaction effect 1,2**	**(6) C.V with main effect 1,2 and interaction effect 1,2,3**	**(7) C.V with main effect 1,2 and interaction effect 1,2,3,4**
*Num*_*of*_*UGC*_*Shares*_*i, t*−1_		0.040^***^	0.041^***^	0.040^***^	0.034^***^	0.030^***^	0.034^***^
*Num*_*of*_*CGC*_*Shares*_*i, t*−1_			0.039^***^	0.039^***^	0.039^***^	0.045^***^	0.037^***^
*Num*_*of*_*UGC*_*Shares*_*i, t*−1_×*Time*_*early*_*t*_				0.013^***^	0.023^***^	0.022^***^	0.017^***^
*Num*_*of*_*UGC*_*Shares*_*i, t*−1_×*Time*_*middle*_*t*_					0.014^***^	0.014^***^	0.008^**^
*Num*_*of*_*CGC*_*Shares*_*i, t*−1_×*Time*_*early*_*t*_						−0.007^***^	−0.002
*Num*_*of*_*CGC*_*Shares*_*i, t*−1_×*Time*_*middle*_*t*_							0.009^***^
*Time*_*early*_*t*_	0.084^***^	0.093^***^	0.105^***^	0.069^***^	0.033^**^	0.066^***^	0.053^***^
*Time*_*middle*_*t*_	0.027^***^	0.035^***^	0.047^***^	0.045^***^	0.002	0.002	−0.020
*Num*_*Of*_*clicks*_*i, t*−1_	0.048^***^	0.050^***^	0.023^***^	0.019^*^	0.019^***^	0.029^***^	0.035^***^
*Num*_*Of*_*videos*_*i, t*−1_	0.182^***^	0.158^***^	0.079^***^	0.072^***^	0.076^***^	0.088^***^	0.082^***^
*Num*_*Of*_*subscribers*_*i, t*−1_	0.062^***^	0.056^***^	0.030^***^	0.032^***^	0.034^***^	0.044^***^	0.043^***^
*Num*_*Of*_*Days*_*After*_*Release*_*i, t*_	−1.345^***^	−1.319^***^	−1.259^***^	−1.263^***^	−1.278^***^	−1.269^***^	−1.260^***^
Constant	1.454^***^	1.401^***^	1.449^***^	1.500^***^	1.552^***^	1.445^***^	1.445^***^
Wald chi-square	302967.21	106844.61	446975.21	371001.81	835038.48	239653.10	287532.86
Number of observations	3,390	3,390	3,390	3,390	3,390	3,390	3,390

* *p < 0.1*,

** *p < 0.05*,

*** *p < 0.01. The dummies of an individual song i used in estimating the model are not reported. Seventy two observations were excluded from Models (4) and (5) because they were represented for only a week (at least 2 weeks' worth of values are needed per each song to estimate covariance of a group controlling for autocorrelation)*.

The first row of Column 7 shows that *Num*_*of*_*UGC*_*Shares*_*i, t*−1_ influences music sales positively and significantly, while the second row of Column 7 shows that *Num*_*of*_*CGC*_*Shares*_*i, t*−1_ influences music sales positively and significantly. *Num*_*of*_*UGC*_*Shares*_*i, t*−1_ and *Num*_*of*_*CGC*_*Shares*_*i, t*−1_ are the proxies of user engagement of UGC and CGC, respectively. In particular, when comparing the effect of user engagement of UGC and CGC on music sales, the effect of user engagement of UGC (0.051) is greater than that of CGC (0.037) in the initial period. However, after the initial period, the effect of user engagement of CGC on music sales becomes similar to that of UGC (e.g., the size of user engagement of CGC on music sales in the middle and final period are 0.046 and 0.037, respectively, while the size of user engagement of UGC are 0.042 and 0.034). Thus, our findings support Hypothesis 1, which states that user engagement of user-generated videos (UGC) has a greater impact on music sales than does the user engagement of company-generated videos (CGC).

These results may indicate that people tend to trust UGC more than CGC in the initial period. Like online word-of-mouth, UGC may be more trustworthy, as it is created by consumers. When people purchase music in the initial period knowing little or nothing about the song, they are more likely to rely on UGC rather than CGC. On the contrary, people may actually regard CGC as an advertisement put out by vendors or music distributors. Therefore, our results show that potential consumers are more likely to purchase music when previous customers have engaged more with UGC than CGC early on.

In addition, we found that the interaction terms, *Num*_*of*_*UGC*_*Shares*_*it*−1_×*Time*_*early*_*t*_ and *Num*_*of*_*UGC*_*Shares*_*it*−1_×*Time*_*middle*_*t*_ have significant and positive coefficients. This suggests that the effect of user engagement of UGC on music sales in the initial and middle periods after a song's release is greater than in the final period. With the main effect (β_1_), the positive effect of user engagement of UGC on music sales is greatest in the initial period (the total effect by adding related coefficients: 0.034 + 0.017 = 0.051); the effect then gradually decreases over the middle (0.034 + 0.008 = 0.042) and final periods (0.034). In short, the effect of user engagement of UGC on music sales is not static but changes through the initial, middle, and final periods after a song's release. Thus, Hypothesis 2-a is supported as we found that the impact of user engagement of user-generated videos (UGC) on music sales is greater during the initial period after a song is released than during the middle and final periods.

We also found that *Num*_*of*_*CGC*_*Shares*_*it*−1_×*Time*_*early*_*t*_ does not have a significant impact on music sales but that *Num*_*of*_*CGC*_*Shares*_*it*−1_×*Time*_*middle*_*t*_ has a significant and positive coefficient. This means that the effect of user engagement of CGC on music sales in the middle period after a song's release is greater than in the final period. There is no difference, however, between the initial and final periods. With the main effect (β_2_), the positive effect of user engagement of CGC on music sales is the most significant in the middle period (the total effect by adding related coefficients: 0.037 + 0.009 = 0.046). The effect sizes in the middle and final period were found to be the same level (0.037). This result does not support Hypothesis 2-b.

To assess the robustness of our research model, we estimated additional models by using various estimation methods (i.e., OLS model, GLS models controlled for both heteroscedasticity and autocorrelation). The results are shown in Table 5. As we see in the table, there were only small changes among the estimations. Since we found heteroscedasticity and first-order autocorrelation in our panel data by the likelihood-ratio test and the Wooldridge test, both the heteroscedasticity and autocorrelation controlled models (Column 5, our research model) offer the most reliable results.

**Table 5:** Robustness.

**Variables**	**(1) OLS estimation**	**(2) GLS estimation (Non-controlled)**	**(3) GLS estimation (heterogeneity-controlled)**	**(4) GLS estimation (Autocorrelation-controlled)**	**(5) GLS estimation (Hetero, auto-controlled)**
*Num*_*of*_*UGC*_*Shares*_*i, t*−1_	0.078^***^	0.078^***^	0.050^***^	0.041^***^	0.034^***^
*Num*_*of*_*CGC*_*Shares*_*i, t*−1_	0.070^***^	0.070^***^	0.055^***^	0.040^***^	0.037^***^
*Num*_*of*_*UGC*_*Shares*_*i, t*−1_×*Time*_*early*_*t*_	0.022^*^	0.022^*^	0.028^***^	0.023^**^	0.017^***^
*Num*_*of*_*UGC*_*Shares*_*i, t*−1_×*Time*_*middle*_*t*_	0.022^**^	0.022^**^	0.019^***^	0.014^*^	0.008^**^
*Num*_*of*_*CGC*_*Shares*_*i, t*−1_×*Time*_*early*_*t*_	−0.003	−0.003	−0.006^**^	0.001	−0.002
*Num*_*of*_*CGC*_*Shares*_*i, t*−1_×*Time*_*middle*_*t*_	0.010	0.01	0.010^***^	0.010^*^	0.009^***^
*Time*_*early*_*t*_	0.086	0.086	0.064^***^	0.028	0.053^***^
*Time*_*middle*_*t*_	−0.017	−0.017	−0.035^**^	−0.042	−0.020
*Num*_*Of*_*clicks*_*i, t*−1_	0.082^***^	0.082^***^	0.067^***^	0.027	0.035^***^
*Num*_*Of*_*videos*_*i, t*−1_	0.058	0.058	0.065^***^	0.095^***^	0.082^***^
*Num*_*Of*_*subscribers*_*i, t*−1_	0.052^***^	0.052^***^	0.063^***^	0.044^***^	0.043^***^
*Num*_*Of*_*Days*_*After*_*Release*_*i, t*_	−1.155^***^	−1.155^***^	−1.215^***^	−1.252^***^	−1.260^***^
Constant	0.953^***^	0.953^***^	1.233^***^	1.340^***^	1.445^***^
Wald chi-square	38.17	19195.2	2.35*e*+07	14610.80	287532.86
Number of observations	3,462	3,462	3,462	3,390	3,390

* *p < 0.1*,

** *p < 0.05*,

*** *p < 0.01, : F-statistics. The dummies of an individual song i used in estimating the model are not reported. Seventy two observations were excluded from Models (4) and (5) because they were represented for only a week (at least 2 weeks' worth of values are needed per each song to estimate covariance of a group controlling for autocorrelation)*.

## Conclusion

The objective of this study was to investigate how user engagement of both user- and company-generated videos influences music sales on YouTube. We found two especially interesting results. First, we compared the effects of user engagement of user-generated content (UGC) and company-generated content (CGC) on music sales. Second, we investigated how these two effects change through the initial, middle, and final periods after a song's release.

We used the number of shares of videos generated by the user and company to measure user engagement of UGC and CGC individually. Using GLS estimation to control for heterogeneity and autocorrelation in our data, we empirically show that both effects of user engagement of UGC and CGC positively influence music sales. We find that the effect of user engagement of UGC is greater than the effect of user engagement of CGC in the initial period. However, after the initial period, the effect of user engagement of CGC on music sales becomes similar to that of user engagement of UGC. This finding emphasizes the importance of user-engagement activities with UGC in the initial period after a product is released.

The findings of this study represent a significant contribution to the academic field. First, although online music video sharing platforms such as YouTube provide access to both UGC and CGC, prior research has not yet examined the impact of user engagement of these two types of music content. This study showed that the way in which users engage with music content is dependent on who created the content and that these ways ultimately lead to different music choices. Such different engagement behaviors may be evidence that user psychology perceives online music content differently depending on who created it. This study contributes to the literature as both UGC and CGC are here considered together to examine how different user engagement effects change over time in ultimately influencing music choices. Second, we suggest a new measurement of user engagement of UGC and CGC by directly collecting online user behavior data. We collected our behavior data from YouTube every week from December 27, 2015 to December 31, 2016 and used the number of shares of UGC and CGC as proxies of user engagement variables (Lee et al., 2013). Thus, this study attempts to measure a psychological dimension—user engagement—with user behavior log data using a novel methodology and show how effects of user engagement change over time. We believe that this study sheds light on novel methodologies that use user behavior log data to collect and measure psychological dimensions. Third, most of the previous studies have focused on user engagement in mass media. We, however, evaluate the effects of user engagement by examining how user-engagement effects play out differently in a social media environment. Fourth, we believe that the effects of user engagement on social media platforms clearly differ in initial, middle, and final periods after a song is released. However, only a few studies have recognized these different dynamics of user-engagement effects across different periods. This research thus investigates the different dynamics of user engagement through the initial, middle, and final periods after a song's release by observing the changing effects of user engagements of UGC and CGC on music sales through the three periods.

The results of this research also hold practical implications. For instance, this study can contribute to the establishment of a marketing strategy for digital music providers by providing insight into how potential consumers are influenced by the two types of social music content-UGC and CGC at different periods. Managers of digital music providers should encourage consumers to create their own content in the initial period, as other potential consumers are more likely to buy songs with more UGC content and shares in the initial period.

This study, however, has several limitations. First, we do not include other variables that may influence music sales. For example, user reviews of music videos, the effects of traditional advertisement in mass media, and recommendations by provider websites could be controlled in future studies. Second, users may engage high quality music videos regardless of who created them. For example, people are more likely to share funny or well-made videos rather than low quality videos created by other users. This study was not able to measure video quality due to the lack of currently available deep learning technology. Third, as the Korean music market is unique in some aspects, such as its idol and fandom culture, our research results may not be applicable worldwide. Fourth, given the limited data collection period, we may have had a song that had no user engagement. Future research may contrast the fate of a song with no user engagement and a song that had user engagement. Such research would enhance the understanding of user engagement. Fifth, the number of total songs we collected (1,035) may not have been enough to generalize our findings to the long term dynamics of user engagement in online social networks. Collecting data for more than 1 year (December 27, 2015 ~ December 31, 2016), however, was infeasible, as we needed to collect data on a weekly basis. We acknowledge this limitation and hope future research will collect more data and show the long term dynamics of user engagement over time. Finally, we hope that future research will examine the impact of various types of user content. Future research should use machine-learning techniques to investigate how different types of user content on social media (text, sound, image, and video) influence user engagement and product sales.

## Author contributions

All authors listed have made a substantial, direct and intellectual contribution to the work, and approved it for publication.

### Conflict of interest statement

The authors declare that the research was conducted in the absence of any commercial or financial relationships that could be construed as a potential conflict of interest.

## Footnotes

^1^Music video content is only one form of social media content. However, we are limited the terms, UGC and CGC, as music video contents for convenience.
